# The Predictive Factors Associated with In-Hospital Mortality of Melioidosis: A Cohort Study

**DOI:** 10.3390/medicina60040654

**Published:** 2024-04-19

**Authors:** Sunee Chayangsu, Chusana Suankratay, Apichat Tantraworasin, Jiraporn Khorana

**Affiliations:** 1 Department of Internal Medicine, Surin Hospital, Surin 32000, Thailand; chayangsu.sunee@gmail.com; 2 Department of Internal Medicine, Faculty of Medicine, The King Chulalongkorn Memorial Hospital, Chulalongkorn University, Bangkok 10330, Thailand; csuankratay@gmail.com; 3 Clinical Surgical Research Center, Department of Surgery, Faculty of Medicine, Chiang Mai University, Chiang Mai 50200, Thailand; apichat.t@cmu.ac.th; 4 Center of Clinical Epidemiology and Clinical Statistic, Faculty of Medicine, Chiang Mai University, Chiang Mai 50200, Thailand; 5 Division of Pediatric Surgery, Department of Surgery, Faculty of Medicine, Chiang Mai University Hospital, Chiang Mai 50200, Thailand

**Keywords:** melioidosis, predictive factors, mortality rate, *Burkholderia pseudomallei*, sepsis, outcome

## Abstract

*Background and Objectives*: Melioidosis is an infectious disease caused by *Burkholderia pseudomallei*, and it has a wide range of clinical symptoms. It is endemic in tropical areas, including Southeast Asia. Despite the availability of effective treatment, the mortality rate is still high, especially in patients presenting with septic shock. The aim of this study was to determine and explore clinical characteristics, microbiology, treatment outcomes, and factors associated with in-hospital mortality which could predict prognosis and provide a guide for future treatment. *Materials and Methods*: The population in this retrospective cohort study included all 262 patients with a diagnosis of melioidosis who were hospitalized at Surin Hospital, Surin, Thailand, from April 2014 to March 2017. We included patients older than 15 years with a positive culture for *B. pseudomallei.* Data regarding the clinical characteristics, microbiology, and treatment outcomes of the patients were collected and analyzed. The patients were divided into two groups dependent on outcome, specifically non-survival and survival. Logistic regression was performed to determine the risk factors associated with in-hospital mortality. *Results*: Out of the 262 patients with melioidosis during the study period, 117 (44.7%) patients died. The mean age was 57.2 ± 14.4 years, and 193 (73.7%) patients were male. The most common comorbidity was diabetes (123, 46.9%), followed by chronic kidney disease (35, 13.4%) and chronic liver disease (31, 11.8%). Four risk factors were found to be associated with in-hospital mortality, including age (adjusted odds ratio (aOR) 1.04, 95%CI: 1.01–1.07), respiration rate (aOR 1.18, 95%CI: 1.06–1.32), abnormal chest X-ray finding (aOR 4.79, 95%CI: 1.98–11.59), and bicarbonate levels (CO_2_) (aOR 0.92, 95%CI: 0.85–0.99). *Conclusions*: Our study identified age, respiration rate, abnormal chest X-ray finding, and CO_2_ levels are predictive factors associated with in-hospital mortality in melioidosis patients. Physicians should be aware of these factors, have access to aggressive treatment options, and closely monitor patients with these risk factors.

## 1. Introduction

Melioidosis is caused by *Burkholderia pseudomallei*, an aerobic, non-spore-forming, non-fermenting Gram-negative bacillus. This endemic organism causes a wide variety of community-acquired infections and is often underreported. It is classified as a class B biological weapon due to its virulent factors. Melioidosis was first reported in 1911 in Yangon, Myanmar, by Whitmore and Krishnaswami [1]. Melioidosis is classified as a neglected tropical disease due to inadequate available data, even though it is an endemic disease in the developing world and the tropics [2]. Melioidosis is endemic in northeast Thailand. The possible routes of entry of infection include direct skin inoculation, droplet transmission, and ingestion. This pathogen can infect any organ of the body, including the skin and soft tissues, joints, bones, the liver, the spleen, the kidney, and the lungs or multiple organs. The lungs are the most common site of infection. Studies from 1997 to 2008 [3] found that the mortality rate of melioidosis was as high as 42.6%, and more recent studies in the literature still cite a very high rate of between 30 and 50% [4]. Our study thus aimed to determine the risk factors associated with mortality in our institute, Surin hospital in northeast Thailand. 

## 2. Materials and Methods

A retrospective cohort study was carried out at Surin Hospital from 1 April 2014 to 31 March 2017. The study was reviewed and approved by the Institutional Ethics Committee under protocol 80/2020. Patients aged of 15 years and over with cultures positive for *B. pseudomallei* were included. Patients with incomplete data or with a final diagnosis that was not melioidosis were excluded. Data collection was carried out using a chart review approach. The two groups consisted of patients with non-survival and survival outcomes, respectively.

Demographic data (sex, age, comorbidity); body mass index (BMI) scores; clinical presentations such as fever; altered sensorium; respiration rate (RR); pulse rate (PR); clinical syndromes; data from blood investigations; and chest X-ray (CXR) findings were collected. Blood laboratory data were taken on the same day blood cultures and/or clinical specimen cultures were taken as part of the septic workup. Vital signs and laboratory data were collected at the same time as the septic work on admission. Abnormal chest X-ray findings were defined as findings related to infiltration, such as the observation of patchy infiltrates, consolidation, reticular findings, ground-glass opacities, and cavitation. Localized infection was defined as 1 site of infection or primary bacteremia without focal infection. Disseminated infection was defined by multi-site infection, specifically more than 1 site of infection with or without bacteremia or 1 site infection with bacteremia. The patients were divided into two groups, survival and non-survival. In-hospital death was defined as a patient who died during admission and included the moribund cases of those who wished to die at home. 

The sample size of this study was calculated based on factors associated with poor outcomes from a previous study [5]. Using alpha 0.05 (two-sided test) and power 0.80, the differences between the mean ± SD of aspartate aminotransferase (AST) in the 2 groups were calculated. The mortality rate was nearly 50%, and to achieve a 1:1 enrollment ratio, the sample required 120 patients in each group.

Statistical analysis was performed using STATA, version 16.0. The collected data are presented as means (standard deviations) when referring to normally distributed data, medians (interquartile ranges) when referring to non-normally distributed data, and counts and percentages when referring to categorical data. Inferential statistics were performed using a *t*-test and the Kruskal–Wallis test for continuous variables and Fisher’s exact test for categorical variables. A *p* value less than 0.05 was considered statistically significant.

Multivariable analysis was performed using logistic regression. The results are reported as odds ratios (ORs) and 95% confidence intervals (CIs). This analysis explored factors associated with in-hospital mortality from melioidosis.

## 3. Results

During the study period, 262 patients were identified, including 117 (44.7%) and 145 (56.3%) patients in the non-survival and survival groups, respectively. Ten patients were excluded due to missing data, and thirteen patients were excluded due to an alternative diagnosis. Demographic and clinical characteristics are shown in Table 1. The non-survival group were significantly older, had a shorter duration of symptoms, had more disseminated or multifocal forms, and had more frequent presentations of cough and altered sensorium. Regarding vital signs, the patients who died had significantly lower systolic blood pressure values, higher pulse rates, and higher respiration rates. The patients who died also had significantly abnormal chest X-rays, elevated neutrophil percentages, blood urea nitrogen, creatinine, aspartate aminotransferase, alanine aminotransferase, and total bilirubin levels. In addition, the patients who died had significantly lower hemoglobin, albumin, and platelet values. Risk factors associated with in-hospital mortality are shown in Table 2. Our univariate analysis identified several factors, including age, duration of symptoms, body mass index, clinical syndromes, vital signs (systolic blood pressure, respiration rate), chest X-ray findings, and laboratory results (platelet count, creatinine, albumin, total bilirubin, aspartate aminotransferase, and bicarbonate level). Overall, 218/262 (83.2%) received an intravenous antibiotic active against *B. pseudomallei*; ceftazidime was the most commonly prescribed antibiotic (151; 57.6%), followed by carbapenems (60; 22.9%), amoxycillin/clavulanic acid (7; 2.7%), and other antibiotics.

**Figure 1:**
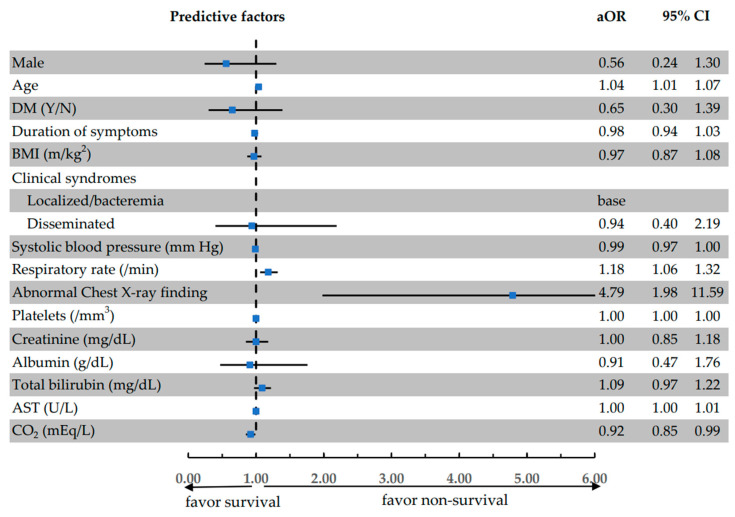
The distributions of the predictive factors for the in-hospital mortality of melioidosis. The odds ratios of final predictors from the multivariable logistic regression are shown. The horizontal error bars indicate 95% confidence intervals. The blue square is adjusted Odd ratio that show in figure too.

A multivariable analysis was performed using odds ratios to identify factors associated with in-hospital mortality (expressed as an adjusted odds ratio (aOR)), including age (aOR 1.04, 95%CI: 0.24–1.30), respiration rate (aOR: 1.18, 95%CI: 1.06–1.32), abnormal chest X-ray findings (aOR 4.79, 95%CI: 1.98–11.59), and serum bicarbonate levels (aOR 0.92, 95%CI: 0.85–0.99). The distributions of the predictive factors are presented in Figure 1, where odds ratios (ORs) and 95% confidence intervals (CIs) are displayed for each factor. As indicated, the factors with an OR greater than 1 are associated with increased mortality risk, while the factors with an OR less than 1 are associated with increased survival.

## 4. Discussion

This study found that the mortality rate of melioidosis was 44.7%, which is in line with previous studies [3,5], suggesting it is still a very critical infectious disease at our institute. In contrast, the recent study by Churuangsak et al. [6] showed a significantly low mortality rate of about 9.0%, probably due to the majority of the patients being categorized as having a localized infection of melioidosis. However, patients with pneumonia and septicemia or shock still carry a high mortality rate risk of 54.2% [5]. Another recent study by Zornitzki et al. [7] found a high rate of positive blood culture or bacteremia even after initiating antibiotic therapy, which may contribute to the high mortality rate. This suggests the complexity of melioidosis diagnosis and treatment. Our study found that most of those in the non-survival group with melioidosis were males aged 50 or older. This age group also had a statistically significant association with mortality, with an odds ratio of 1.04. In other words, for every year older a patient was, their risk of death from melioidosis increased by 4%. These findings are consistent with previous studies suggesting that melioidosis is more common in older individuals, particularly farmers who frequently work in rice fields. This increased risk is likely due to their higher exposure to contaminated soil and water, which harbor the bacteria *Burkholderia pseudomallei*, the causative agent of melioidosis [3,8,9,10,11]. In this study, 80% of infected individuals had at least one comorbidity, with diabetes mellitus being the most common, a finding in line with previous studies [2,12]. Nevertheless, it is worth noting that in this study, the presence of diabetes mellitus as a comorbidity was not found to have a significant association with mortality, in contrast to previous studies [13,14,15,16]. In fact, diabetes mellitus appears to be a protective factor against mortality in melioidosis (OR 0.65; 95%CI 0.30–1.39). This finding is of interest, but it is not statistically significant. This may be due to the fact that diabetic patients presenting with sepsis in the clinical setting in this institute are initially treated with antimicrobials that cover *B. pseudomallei* due to the disease’s endemic nature [17,18]. Treatment with carbapenems (meropenem or imipenem) was more frequent in the non-survival group. This could indicate that carbapenems are usually used for treating serious infections. 

The majority of patients were categorized as having disseminated or multifocal forms of melioidosis, which resulted in a high mortality rate. Most patients who died presented with a shorter duration of symptoms than those who survived, indicating the severity of the presenting condition, a finding consistent with previous studies [5,6]. Furthermore, the non-survival group had a higher prevalence of abnormalities in chest X-rays, as well as elevated levels of %PMN, blood urea nitrogen, creatinine, aspartate aminotransferase, alanine aminotransferase, globulin, total bilirubin, and direct bilirubin. Additionally, this group showed reduced platelet counts and lower levels of albumin and bicarbonate when compared to those who survived. 

In addition to the established association between increasing age and fatal melioidosis, the multivariate analysis revealed several other significant factors. Notably, a 1 min increase in respiration rate was associated with an 18% increase in mortality risk (95%CI 1.06–1.32). Furthermore, abnormal CXR findings demonstrated the highest OR value of 4.79 (95%CI 1.98–11.59), suggesting a strong correlation with pulmonary involvement as a major factor contributing to increased mortality in melioidosis patients, and a low level of bicarbonate may indicate severe sepsis, consistent with previous studies [5,19,20,21]. The study also found that AST levels may be a potential predictive factor, although the odds ratio for this was 1, and the *p*-value was below 0.05, so this remains inconclusive. Studies with larger sample sizes are necessary to validate the relationship between AST levels and fatal melioidosis. 

There are limitations to this study. The retrospective nature of the study inevitably resulted in some missing data, and the standard severity scores from systems such as APACHE II and SOFA could not be obtained for all patients. In addition, long-term mortality was not analyzed since all infections, including pneumonia and sepsis, can lead to inflammation for more than 6 months. However, the aim of this study was just to determine the risk factors associated with short-term mortality. The value of this study is that the findings have the potential to inform physicians about mortality-associated factors in melioidosis, enabling the creation of clinical prediction guidelines which will facilitate optimized treatments for such a deadly condition. In the future, grouping patients based on severity of illness or mortality rate may help guide prospective studies on the selection of antimicrobial therapy. This could involve using combination antibiotics (carbapenem plus trimethoprim–sulfamethoxazole (TMP-SMX) or ceftazidime plus TMP-SMX) or carbapenems as a first-line treatment.

## 5. Conclusions

In summary, there is still a very high mortality rate among melioidosis patients in our institute despite effective antimicrobial treatment. Most patients who died presented in the critical condition, confirmed by severe sepsis, shock, and multi-organ involvement. Educating the people at risk, especially farmers, regarding the symptoms of suspected melioidosis could be the mainstay in preventing late presentation and ultimately reducing in-hospital mortality. The factors associated with high mortality in melioidosis are age, respiration rates, chest X-ray abnormalities, and serum aspartate aminotransferase and serum bicarbonate levels. Focused and cautious assessments of patients with melioidosis from intensive care settings that consider the use of antimicrobials for high-risk patients are of the utmost importance.

## Figures and Tables

**Table 1:** Baseline demographic and clinical characteristics of patients with melioidosis in the non-survival and survival groups.

Characteristics	Non-Survival (n = 117)	Survival (n = 145)	*p*-Value
Male ^1^	83 (70.4%)	110 (75.9%)	0.399
Age (years) ^2^	59.3 ± 15.7	55.5 ± 13.2	0.037
Comorbidities (Y/N) ^1^	93 (79.5%)	116 (80.0%)	1.000
DM ^1^	49 (41.9%)	74 (51.0%)	0.171
Chronic kidney disease ^1^	18 (15.4%)	17 (11.7%)	0.466
Cirrhosis ^1^	15 (12.8%)	16 (11.0%)	0.703
Duration of symptoms (days) ^3^	4 (3–7)	7 (3–14)	<0.001
Clinical syndromes ^1^			<0.001
Localized or bacteremia *	35 (29.9%)	77 (53.1%)	
Disseminated or multifocal	82 (70.1%)	68 (46.9%)	
Fever ^1^	106 (90.6%)	125 (86.2%)	0.337
Cough ^1^	54 (46.2%)	41 (28.3%)	0.003
Altered sensorium ^1^	20 (17.1%)	9 (6.2%)	0.009
BMI (kg/m^2^) ^2^	20.6 ± 3.7	21.7 ± 3.7	0.026
Systolic blood pressure (mm Hg) ^2^	117.1 ± 27.7	125.8 ± 23.3	0.006
Pulse rate (per minute) ^2^	107.0 ± 24.9	97.0 ± 18.2	<0.001
Respiration rate (per minute) ^2^	25.2 ± 4.9	21.7 ± 2.7	<0.001
Abnormal chest X-ray ^1^	98 (84.5%)	64 (44.4%)	<0.001
%Neutrophils ^2^	85.9 ± 10.7	81.4 ± 10.7	<0.001
HCT (%) ^2^	31.3 ± 7.9	33.1 ± 7.1	0.061
Hb (g/dL) ^2^	10.2 ± 2.6	10.8 ± 2.3	0.049
MCV (fL) ^2^	81.1 ± 9.9	80.5 ± 8.7	0.608
Platelets (/mm^3^) ^3^	165,000 (93,000–219,000)	213,000 (159,000–327,000)	<0.001
BUN (mg/dL) ^3^	37 (22–59)	17 (12–28)	<0.001
Creatinine (mg/dL) ^3^	1.9 (1.2–3.4)	1.1 (0.8–1.6)	<0.001
AST (U/L) ^3^	107 (63–258)	53.5 (35–93)	<0.001
ALT (U/L) ^3^	56.5 (26–126)	41 (23–67)	0.002
Albumin (g/dL) ^2^	2.5 ± 0.6	2.8 ± 0.6	<0.001
Globulin (g/dL) ^2^	3.4 ± 0.7	3.7 ± 0.6	0.002
Total bilirubin (mg/dL) ^3^	1.7 (0.9–4.0)	0.9 (0.5–1.7)	<0.001
Direct bilirubin (mg/dL) ^3^	1.0 (0.5–2.7)	0.3 (0.1–0.8)	<0.001
Na (mEq/L) ^2^	130.2 ± 6.5	130.1 ± 6.0	0.936
CO_2_ (mEq/L) ^2^	17.7 ± 6.5	22.3 ± 5.6	<0.001

DM = diabetic mellitus; BMI = body mass index; HCT = hematocrit; Hb = hemoglobin; MCV = mean corpuscular volume; BUN = blood urea nitrogen; AST = aspartate aminotransferase; ALT = alanine aminotransferase; Na = sodium; CO_2_ = bicarbonate; * localized form—negative growth on blood culture with the involvement of one organ; bacteremia—positive growth on blood culture with no identified organ involvement. ^1^ reported as count or percentage and analyzed using Fisher’s exact test. ^2^ reported as mean ± standard deviation and analyzed using Student’s *t*-test. ^3^ reported as median and interquartile range and analyzed using the Kruskal–Wallis test.

**Table 2:** Multivariable risk ratios of predictive factors for in-hospital mortality in melioidosis patients.

Predictive Factors	Univariable Odds Ratio (95% Confidence Interval)	*p*-Value	Multivariable Odds Ratio (95% Confidence Interval)	*p*-Value
Male	0.78 (0.45–1.35)	0.369	0.56 (0.24–1.30)	0.176
Age	1.02 (1.00–1.04)	0.039	1.04 (1.01–1.07)	0.019
DM (Y/N)	0.69 (0.42–1.13)	0.141	0.65 (0.30–1.39)	0.267
Duration of symptoms	0.96 (0.93–0.99)	0.005	0.98 (0.94–1.03)	0.398
BMI (m/kg^2^)	0.92 (0.86–0.99)	0.028	0.97 (0.87–1.08)	0.532
Clinical syndromes				
Localized/bacteremia	base		base	
Disseminated	2.65 (1.59–4.43)	<0.001	0.94 (0.40–2.19)	0.885
Systolic blood pressure (mm Hg)	0.99 (0.98–1.00)	0.007	0.99 (0.97–1.00)	0.142
Respiration rate (/min)	1.33 (1.21–1.47)	<0.001	1.18 (1.06–1.32)	0.003
Abnormal chest X-ray finding ^1^	6.81 (3.74–12.41)	<0.001	4.79 (1.98–11.59)	0.001
Platelets (/mm^3^)	1.00 (1.00–1.00)	<0.001	1.00 (1.00–1.00)	0.432
Creatinine (mg/dL)	1.24 (1.09–1.42)	<0.001	1.00 (0.85–1.18)	0.995
Albumin (g/dL)	0.43 (0.28–0.66)	<0.001	0.91 (0.47–1.76)	0.778
Total bilirubin (mg/dL)	1.20 (1.07–1.34)	0.001	1.09 (0.97–1.22)	0.157
AST (U/L)	1.01 (1.00–1.01)	<0.001	1.00 (1.00–1.01)	0.016
CO_2_ (mEq/L)	0.88 (0.84–0.92)	<0.001	0.92 (0.85–0.99)	0.036

^1^ an abnormal chest X-ray finding was defined as findings related to infiltration, such as the observation of patchy infiltrates, consolidation, reticular findings, ground-glass opacities, and cavitation.

## Data Availability

The datasets used during the current study are available from the corresponding author on reasonable request.
